# Genetic Variants Associated with Non-Alcoholic Fatty Liver Disease Do Not Associate with Measures of Sub-Clinical Atherosclerosis: Results from the IMPROVE Study

**DOI:** 10.3390/genes11111243

**Published:** 2020-10-22

**Authors:** Luigi Castaldo, Federica Laguzzi, Rona J. Strawbridge, Damiano Baldassarre, Fabrizio Veglia, Lorenzo Vigo, Elena Tremoli, Ulf de Faire, Per Eriksson, Andries J. Smit, Jiri Aubrecht, Karin Leander, Matteo Pirro, Philippe Giral, Alberto Ritieni, Giovanni Di Minno, Anders Mälarstig, Bruna Gigante

**Affiliations:** 1 Department of Clinical Medicine and Surgery, University of Naples “Federico II”, 80138 Naples, Italy; giovanni.diminno@unina.it; 2 Department of Pharmacy, University of Naples “Federico II”, 80138 Naples, Italy; alberto.ritieni@unina.it; 3 Unit of Cardiovascular and Nutritional Epidemiology, Institute of Environmental Medicine, Karolinska Institutet, Box 210, SE-171 77 Stockholm, Sweden; federica.laguzzi@ki.se (F.L.); Ulf.deFaire@ki.se (U.d.F.); Karin.Leander@ki.se (K.L.); 4 Mental Health and Wellbeing, Institute of Health and Wellbeing, University of Glasgow, Glasgow G12-8QQ, UK; Rona.Strawbridge@glasgow.ac.uk; 5 Health Data Research University of Glasgow, College of Medicine, Veterinarian and Life Sciences, Glasgow G12-8RZ, UK; 6 Cardiovascular Medicine, Department of Medicine, Karolinska Institutet, Box 210, 171 77 Stockholm, Sweden; Per.Eriksson@ki.se (P.E.); anders.malarstig@ki.se (A.M.); bruna.gigante@ki.se (B.G.); 7 Centro Cardiologico Monzino, Istituti di Ricovero e Cura a Carattere Scientifico (IRCCS), Via Parea 4, 20138 Milan, Italy; Damiano.Baldassarre@cardiologicomonzino.it (D.B.); Fabrizio.Veglia@cardiologicomonzino.it (F.V.); lorenzo.vigo@cardiologicomonzino.it (L.V.); elena.tremoli@cardiologicomonzino.it (E.T.); 8 Department of Medical Biotechnology and Translational Medicine, University of Milan, 20122 Milano MI, Italy; 9 Department of Medicine, Division of vascular medicine University Medical Center Groningen, 9713 GZ Groningen, The Netherlands; a.j.smit@umcg.nl; 10 Takeda Pharmaceuticals International Co., Cambridge, 02139 MA, USA; jiri.aubrecht@pfizer.com; 11 Unit of Internal Medicine, Department of Medicine, University of Perugia, 06123 Perugia PG, Italy; matteo.pirro@unipg.it; 12 Assistance Publique—Hopitaux de Paris; Service Endocrinologie-Metabolisme, Groupe Hôpitalier Pitie-Salpetriere, Unités de Prévention Cardiovasculaire, 75013 Paris, France; philippe.giral@psl.aphp.fr

**Keywords:** carotid intima-media thickness, non-alcoholic fatty liver disease, alanine aminotransferase and genetic association study

## Abstract

Non-alcoholic fatty liver disease (NAFLD) and atherosclerosis-related cardiovascular diseases (CVD) share common metabolic pathways. We explored the association between three NAFLD-associated single nucleotide polymorphisms (SNPs) rs738409, rs10401969, and rs1260326 with sub-clinical atherosclerosis estimated by the carotid intima-media thickness (c-IMT) and the inter-adventitia common carotid artery diameter (ICCAD) in patients free from clinically overt NAFLD and CVD. The study population is the IMPROVE, a multicenter European study (*n* = 3711). C-IMT measures and ICCAD were recorded using a standardized protocol. Linear regression with an additive genetic model was used to test for association of the three SNPs with c-IMT and ICCAD. In secondary analyses, the association of the three SNPs with c-IMT and ICCAD was tested after stratification by alanine aminotransferase levels (ALT). No associations were found between rs738409, rs1260326, rs10401969, and c-IMT or ICCAD. Rs738409-G and rs10401969-C were associated with ALT levels (*p* < 0.001). In patients with ALT levels above 28 U/L (highest quartile), we observed an association between rs10401969-C and c-IMT measures of c-IMT_max_ and c-IMT_mean-max_ (*p* = 0.018 and 0.021, respectively). In conclusion, NAFLD-associated SNPs do not associate with sub-clinical atherosclerosis measures. However, our results suggest a possible mediating function of impaired liver function on atherosclerosis development.

## 1. Introduction

The mechanisms underlying the mutual interaction between the liver and the heart are poorly investigated [1]. Given the high prevalence in the population of both liver and cardiac diseases, understanding the factors associated with an increased risk of cardiovascular diseases (CVD) in individuals with impaired liver function, and vice-versa, is highly clinically relevant [2].

Non-alcoholic fatty liver disease (NAFLD) affects about 20–30% of the population [3] and share several risk factors with atherosclerosis [4]. However, a causal association between these two conditions has not been demonstrated [5]. Atherosclerosis is a chronic inflammatory disease of the vessel wall associated with a plethora of clinical manifestations in different vascular beds. Coronary heart disease (CHD) and atherothrombotic stroke, the two most common atherosclerotic related CVD, represent the first cause of death in Western countries [6].

NAFLD is characterized by lipid accumulation in the liver (hepatic steatosis) in the absence of alcohol consumption and increased circulating levels of the hepatic enzyme alanine aminotransferase (ALT). Hepatic steatosis has been associated with components of the metabolic syndrome (abdominal obesity, insulin resistance, hypertriglyceridemia) [7] and low grade systemic chronic inflammation [8], all factors known to predispose to the development of atherosclerosis in the vessel wall.

Genetic variants in three genes involved in lipid and glucose metabolism, rs738409 (C/G) in the patatin-like phospholipase domain-containing 3 (*PNPLA3*) gene, rs10401969 (T/C) in the transmembrane 6 superfamily member 2 (*TM6SF2*) gene, and rs1260326 (C/T) in the glucokinase regulatory protein (*GCKR*) gene, have been consistently associated with the risk of NAFLD and NAFLD hepatic complications. No association was reported with the risk of CVD [5,9], while the association with cardiometabolic traits is controversial [10].

Epidemiological studies have been mostly performed so far in patients diagnosed with NAFLD. Overall, NAFLD patients with elevated ALT circulating levels have more frequent carotid atherosclerotic plaques, a higher risk of CHD [11,12,13], an increased prevalence of sub-clinical atherosclerosis, measured by carotid intima-media thickness (c-IMT), reduced arterial distensibility, and increased coronary artery calcium [14,15].

Identifying individuals at highest CVD risk represents an important step to improve CVD prevention and treatment. In the present study, we sought to investigate the association of these three NAFLD-associated genetic variants, rs738409, rs10401969, and rs1260326, with measures of sub-clinical atherosclerosis in a large European cohort consisting of individuals without overt NAFLD and free of clinical CVD manifestation. As measures of carotid sub-clinical atherosclerosis, we used c-IMT and inter-adventitia common carotid artery diameter (ICCAD), both correlated with coronary atherosclerosis [16]. In secondary analysis, we have estimated the association of the aforementioned single nucleotide polymorphisms (SNPs) with c-IMT and ICCAD stratified by circulating ALT levels.

## 2. Materials and Methods

### 2.1. Study Population

We performed our study in a large European cardiovascular cohort including individuals at high risk of cardiovascular events (acronym: IMPROVE (Carotid **I**ntima **M**edia Thickness and IMT-**PR**ogression as Predictors of **V**ascular **E**vents in a High-Risk European Population)). The IMPROVE study was previously described [16]. Briefly, between 2004 and 2005, 3711 participants, free from CVD, but with at least three conventional atherosclerosis risk factors, were recruited in five European countries. Study participants reported their lifestyle habits, previous and current diseases, and medications, and underwent a physical examination. Blood samples were withdrawn and stored at −80 °C in the biobank. Height (m) and weight (kg) were used to estimate body mass index (BMI; kg/m^2^). Hypertension was defined as blood pressure higher than 140/90 mmHg at the visit and/or if self-reported and/or in the presence of treatment; diabetes was defined if blood glucose ≥ 7.0 mmol/L and/or if self-reported and/or in the presence of treatment with glucose-lowering drugs or insulin. Smoking was defined either as never and former or as current smoking. Plasma total cholesterol (TC), LDL-cholesterol (LDL-C), triglycerides (TG), glucose level, and ALT were measured using standard enzymatic methods from fasting blood samples [16].

### 2.2. Ethics

The study was designed following the rules of Good Clinical Practice (GCP) and with the ethical principles established in the Declaration of Helsinki. Each participant provided two different informed consents; one for general participation in the study and one for genotyping. The regional ethical committee at Karolinska Institutet has approved the study (Dnr 2003-115, 2017/404-32, 2019/06387).

### 2.3. Ultrasonographic Measures

The carotid artery ultrasonographic scan was recorded at baseline measuring four consecutive segments at the far wall of the left and right carotid artery in three angles (anterior, lateral, and posterior). Data from the eight segments measured in each patient were averaged to estimate the c-IMT_mean_, c-IMT_max_, and c-IMT_mean-max_. The inter-adventitia common carotid artery diameter (ICCAD) was measured in a plaque free area of the second centimeter of the common carotid proximal to the bifurcation. Data are expressed in mm. Details on protocol, validation, and precision of carotid ultrasound measurements were previously described [17].

### 2.4. Genotyping

Genomic DNA from IMPROVE study participants was genotyped with two genotyping arrays, the CardioMetaboChip 200 K and the Immunochip, each one analyzing approximately 200,000 genetic variants [18]. The CardioMetaboChip 200 K is a custom Illumina iSelect genotyping. The Immunochip is a custom Illumina Infinium HD array designed to densely genotype immune-mediated diseases using loci identified by genome-wide association (GWA) studies. Standard quality control procedures for genetic data were conducted on the individual genotyping chips as well as the combined chip (CardioMetabo-Immuno, Metabochip, Illumina, San Diego, CA, USA) [19]. Measurements of DNA concentration, standardization of DNA concentration in DNA samples, aliquoting, and plating were performed according to a pre-established standard operating procedures (SOP). One aliquot has been stored at −20 °C for long-term use, with a diluted working stock kept in a microtiter array.

Multidimensional scaling (MDS) components were calculated using PLINK version 1.07 [20] (using default settings) to identify possible non-European ethnicity and used to adjust for population stratification. SNPs were excluded for deviation from Hardy–Weinberg equilibrium (*p* < 0.0000001), call rate < 95%, or minor allele frequency (MAF) < 1%. Subjects with call rate < 95%, cryptic relatedness, ambiguous sex, or identified as outliers by MDS were also excluded. Genotype data for rs738409 (C/G), rs10401969 (T/C), and rs1260326 (C/T) were extracted from the CardioMetabo-Immuno chip after the quality control procedures described above.

After exclusion of study participants with no visualized carotid artery (*n* = 7) and missing genotypes (*n* = 356), a total of 3347 subjects were analyzed in the present study. Given the low allele frequency of the effect allele (EA) for rs10401969, heterozygous CT and homozygous CC (*n* = 393 and *n* = 19, respectively) were pooled in the analysis.

### 2.5. Statistical Analysis

Continuous variables were expressed as median and interquartile range (IQR) and categorical variables as percentages. Linear regression models were used to assess the association between the studied SNPs, rs738409 (C/G), rs1260326 (C/T), and rs10401969 (T/C), and c-IMT measures (c-IMT_mean_, c-IMT_max_, and c-IMT_mean-max_), ICCAD, ALT, and metabolic traits including BMI, TC, LDL-C, TG, and glucose level. Individuals with type 2 diabetes were excluded from the analysis of glucose levels. C-IMT measures and ICCAD were log transformed when pertinent to achieve a normal distribution. Analyses were performed assuming an additive model of inheritance. Results are reported as β (β) coefficient and standard error (SE). Estimates were adjusted for age, sex, and multidimensional scaling dimensions (MSD). Three MSD components were found to be informative (MSD1, MSD2, MSD3) and introduced in the analytical model, as previously reported [21]. In secondary analyses, ALT (U/L) levels were categorized in quartiles (1st quartile (Q), Q1: ≤16, *n* = 876; Q2: >16–≤21, *n* = 929; Q3: >21–≤28, *n* = 801; Q4: >28, *n* = 741). The association of the three SNPs with c-IMT and ICCAD levels in the different ALT quartiles was then estimated by linear regression, after adjustment for age, gender, and MSD1–3.

Multiple testing correction was not applied for ultrasonographic measures because they are strongly correlated. However, in the main analysis we corrected for multiple comparison considering 5 independent tests (c-IMT and ICCAD, lipids, ALT, BMI, and glucose). All statistical tests were two-sided and a *p* < 0.01 was then considered statistically significant. Statistical analysis was performed using STATA 12 (StataCorp LP, College Station, TX, USA).

## 3. Results

### 3.1. Clinical Characteristics

Clinical, biochemical, and ultrasonographic characteristics of the study participants by rs738409 (C/G), rs10401969 (T/C), and rs1260326 (C/T) genotypes are presented in Table 1.

Study participants carrying the GG genotype at rs738409 had higher TC, LDL-C, and ALT levels and lower c-IMT and ICCAD when compared to the CC and CG genotype groups. Those carrying the TC/CC genotype of rs10401969 had higher glucose, LDL-C, ALT, c-IMT, and ICCAD values and lower TC and TG levels as compared to the CC group. Finally, TT carriers at rs1260326 had lower BMI, TG, ALT, c-IMT, and ICCAD values and higher TC and LDL-C levels as compared to the CC/CT carriers.

### 3.2. Association of rs738409 (C/G), rs10401969 (T/C), and rs1260326 (C/T) with c-IMT and ICCAD

No associations were observed between the effect allele (EA) rs738409-G, rs10401969-C, and rs1260326–T (vs. the respective common allele) with C-IMT and ICCAD, as shown in Table 2.

### 3.3. Association of rs738409 (C/G), rs10401969 (T/C), and rs1260326 (C/T) with Metabolic Traits

Table 3 shows the results of the association of EA for each of the SNPs with ALT and metabolic traits. In multivariate analysis, rs738409-G and rs10401969-C were associated with ALT circulating levels (β = 0.029, SE = 0.006, *p* < 0.001 and β = 0.040, SE = 0.009, *p* < 0.001, respectively), while no association was observed for rs1260326-T. When we tested the association of the three SNPs with metabolic traits, only rs1260326-T was found to be associated with TG (β = 0.105, SE = 0.020, *p* < 0.001) and BMI (β = −0.224, SE = 0.102, *p* = 0.028).

### 3.4. Association of rs738409 (C/G), rs10401969 (T/C), and rs1260326 (C/T) with c-IMT and ICCAD after Stratification by ALT Levels

Supplementary Table S1 shows c-IMT measures and ICCAD across rs738409, rs10401969, and rs1260326 genotype groups stratified by ALT quartiles. As shown in Supplementary Table S2, rs10401969-C was positively associated with c-IMT_max_ and c-IMT_mean-max_ (*p* = 0.018 and 0.021, respectively) in the highest ALT quartile. No significant associations between rs738409 and rs1260326 and c-IMT and ICCAD were observed in any of the ALT quartiles.

## 4. Discussion

The main finding of our study is that genetic variants consistently associated with NAFLD were not associated with measures of sub-clinical atherosclerosis (c-IMT and ICCAD) in European individuals at high CV risk without overt CVD and NAFLD. Our results confirm and extend previous observations showing an association of rs738409 and rs10401969 with ALT circulating levels and suggest that atherosclerosis burden might be higher in the presence of rs10401969-C and high ALT levels. Consistent with this, our data support the hypothesis that an impaired liver function might contribute to, or be associated with, more severe atherosclerotic disease [22,23].

Rs738409 (C/G) maps at the patatin-like phospholipase domain-containing 3 (*PNPLA3*) gene, encoding an enzyme with lipid acyl hydrolase activity. Rs738409-G causes an amino acid substitution (I148M) in a *PNPLA3* side chain, able to modify the catalytic properties of the enzyme leading to triglyceride accumulation in the liver [24]. Moreover, PNPLA3 I148M seems to disrupt enzyme activity, which leads to a reduced incorporation of triglycerides into very low density lipoprotein, resulting in the increase intracellular fat content [25]. The increased hepatic fat accumulation observed in subjects carrying the rs738409-G allele may explain the elevated ALT levels [25,26].

Studies performed in NAFLD patients have provided controversial results on the association between rs738409 (C/G) and measures of sub-clinical atherosclerosis [27,28]. In young NAFLD patients, rs738409-GG was associated with a greater severity of carotid plaques, thicker c-IMT, and with c-IMT progression [27]. On the other hand, rs738409-GG was found to be associated with c-IMT measures only in subjects with metabolic syndrome and NAFLD [28] and was not associated with c-IMT measures in a Chinese cohort of 4300 middle-aged men and women [29].

Our findings do not support an association of this SNP either with measures of sub-clinical atherosclerosis or with metabolic traits, thus supporting the hypothesis that this SNP is not mainly related to CVD risk, but to NAFLD risk.

Rs10401969 maps to a locus at Chr19p13.11 [30], known to be of importance in the regulation of lipid metabolism [31]. Previous studies have suggested that *TM6SF2*, a gene expressed in the small intestine and in the liver and involved in regulation of lipid absorption and metabolism [32], is the causative gene within the Chr19p13.11 locus [33]. Rs10401969 is in strong linkage disequilibrium (LD) in European populations with a nonsynonymous mutation where presence of rs58542926-G replaces a glutamate with lysine at residue 167 (E167K) [34]. This amino-acid change causes misfolding and an accelerated degradation of *TM6SF2* that results in reduced lipid absorption and hepatic metabolism. This predisposes to liver steatosis and related hepatic complications and at the same time maintains a favorable, non-atherogenic lipid profile consistent with the observation that this mutation exerts a protective effect towards CVD [35,36]. Here, we found an indication of a consistent effect of rs10401969-C on transaminase levels and sub-clinical atherosclerosis, suggesting that individuals carrying a genetic risk of developing NAFLD and high circulating transaminase levels have a more pronounced sub-clinical atherosclerosis. While it is difficult to directly compare our study with those previously published since our population has a very high risk of CVD, our results confirm and extend previous knowledge on the metabolic intertwine between NAFLD and atherosclerosis. In line with our observations, ALT levels were associated with an increased risk of CVD in a Dutch population after adjustment for determinants of the metabolic syndrome and other CV risk factors [37], and with c-IMT in Korean patients with different degrees of NAFLD [38]. These results may prompt additional clinical investigations and underscore the importance of a careful evaluation about the initiation of strict cardiovascular prevention measures in individuals with elevated transaminase levels and high CVD risk.

Rs1260326-T at *GCKR* causes an amino-acid change (P446L) in the glucokinase regulatory protein and it was recognized as an important determinant of inter-individual variation in liver fat [39,40]. *GCKR* modulates the activity of glucokinase, an enzyme involved in the regulation of glucose metabolism and storage in the liver. As widely reported in population-based studies, GCKR represents a genetic factor linked to serum cholesterol and triglyceride levels [41]. However, Varbo et al. [42] evaluate the association between GCKR-rs1260326 with lipid levels and risk of ischemic heart disease (IHD) and myocardial infarction (MI) in the general population, reporting that GCKR-rs1260326 did not influence low-density lipoprotein cholesterol levels or risk of IHD or MI. Presence of the amino-acid substitution has been reported to be associated with a favorable effect on glucose and insulin metabolism, but with an increased biosynthesis of lipids in the liver [43]. This SNP has been formerly associated with a thicker c-IMT in patients with metabolic syndrome [44], but was not associated with c-IMT measures in individuals without myocardial infarction or type 2 diabetes [30]. Moreover, rs780094 in the *GCKR* locus, in strong LD with rs1260326, was also associated with a significant increase in c-IMT in men but not in women in a general population Japanese cohort [45]. We have not observed an association of this SNP with ALT or c-IMT in our study, but a favorable effect on BMI and triglyceride levels.

This study has several strengths: a large sample size, a wide range of important vascular risk factors, consistent methodology for a large number of ultrasonographic measures; standardized methods for carotid image acquisition. Limitations of our study are mainly related to the lack of assessment of liver pathology. Fatty liver was not defined using some imaging modality or biopsies. However, we used three SNPs consistently associated with NAFLD and transaminase levels as a proxy for NAFLD and impairment of liver function. Moreover, other factors possibly modifying ALT, such as chronic hepatitis B or C, autoimmune hepatitis, α-1 antitrypsin deficiency, drug-associated, hemochromatosis, Wilson disease, ischemic hepatitis, and Budd-Chiari syndrome, were not assessed.

## 5. Conclusions

Our study shows no association of genetic variants associated with NAFLD and measures of sub-clinical atherosclerosis and confirms and extends previous knowledge on the lack of causality in the association between these two largely prevalent conditions. At the same time, our results suggest that in selected groups at risk for CVD, elevated ALT may exacerbate sub-clinical atherosclerosis. This may imply that a certain group of patients characterized by elevated ALT levels and high CVD risk may benefit from more aggressive CVD treatment and prevention.

## Figures and Tables

**Table 1:** Clinical characteristics, and biochemical and ultrasonographic measurements in the IMPROVE study population stratified by rs738409 (C/G), rs10401969 (T/C), and rs1260326 (C/T). Data are expressed as median (interquartile ranges) for continuous variables or number (percent) for categorical variables.

		rs738409			rs10401969		rs1260326		
	*n*	CC	CG	GG	TT	CT + CC	CC	CT	TT
Men, *n* (%)	3347	1070 (47.9)	482 (48.9)	46 (42.6)	1385 (47.2)	221(53.6)	556 (52.0)	712 (45.0)	389 (53.4)
Age, years	3347	65.0 (59.8–67.3)	64.2 (59.6–67.1)	62.21 (58.6–66.6)	64.3 (59.5–67.2)	66.3 (60.8–67.2)	64.6 (59.5–67.1)	65.0 (60.1–67.4)	63.8 (59.4–63.3)
BMI, (kg/m^2^)	3346	26.8 (24.2–29.3)	26.9 (24.6–29.7)	26.7 (24.1–30.9)	26.8 (24.2–29.4)	26.8 (24.6–29.8)	27.28 (24.7–30.2)	26.7 (24.2–29.1)	24.5 (23.8–28.9)
**Cardiovascular Risk Factors, *n* (%)**									
Current smoke	3347	351 (15.6)	135 (13.7)	16 (14.8)	448 (15.6)	54 (13.1)	163 (15.2)	223 (14.4)	116 (15.9)
Diabetes mellitus Type 2	3294	570 (25.7)	267 (27.5)	31 (29.8)	751 (26.0)	117 (28.7)	333 (31.7)	382 (25.11)	153 (21.2)
Hypertension	3347	1787 (79.28)	789 (80.1)	85 (78.7)	2310 (78.7)	351 (85.2)	881 (82.3)	1218 (78.63)	562 (77.2)
**Biochemical Measurements**									
TC	3341	5.4 (4.7–6.2)	5.4 (4.7–6.2)	5.6 (4.5–6.4)	5.5 (4.7–6.2)	5.2 (4.6–6.0)	5.3 (4.6–6.2)	5.4 (4.7–6.2)	5.6 (4.9–6.3)
LDL-C	3347	3.5 (2.8–4.2)	3.5 (2.8–4.2)	3.6 (2.7–4.5)	3.5 (2.8–4.2)	3.4 (2.9–4.1)	3.5 (2.8–4.2)	3.5 (2.8–4.2)	3.64 (3.0–4.3)
HDL-C	3341	1.2 (1.0–1.5)	1.2 (1.0–1.4)	1.2 (1.0–1.4)	1.2 (1.0–1.5)	1.2 (1.0–1.4)	1.2 (1.0–1.5)	1.2 (1.0–1.5)	1.2 (1.0–1.5)
TG	3341	1.3 (0.9–1.9)	1.3 (0.9–1.9)	1.3 (0.9–1.7)	1.3 (0.9–1.9)	1.2 (0.9–1.7)	1.2 (0.9–1.7)	1.3 (0.9–1.8)	1.4 (1.0–2.1)
Glucose	3341	5.5 (4.9–6.3)	5.5 (5.0–6.4)	5.4 (4.9–6.4)	5.5 (4.9–6.3)	5.6 (5.1–6.6)	5.6 (5.1–6.5)	5.5 (5.0–6.3)	5.4 (4.8–6.0)
ALT	3347	20 (16.0–27.0)	21 (17.0–29.0)	23.5 (17.5–34.5)	20 (16–27)	22 (17.0–30.0)	21 (17.0–29.0)	20 (16.0–27.0)	20 (17.0–27.0)
**Ultrasonographic Measures (mm)**									
C-IMT_mean_	3346	0.85 (0.7–1.0)	0.85 (0.7–1.0)	0.81 (0.7–0.9)	0.85 (0.7–1.0)	0.87 (0.8–1.0)	0.86 (0.7–1.0)	0.85 (0.7–1.0)	0.84 (0.7–1.0)
C-IMT_max_	3346	1.85 (1.4–2.5)	1.93 (1.4–2.6)	1.84 (1.4–2.3)	1.85 (1.4–2.5)	1.93 (1.4–2.5)	1.93 (1.4–2.6)	1.85 (1.4–2.5)	1.84 (1.4–2.4)
C-IMT_mean-max_	3347	1.19 (1.0–1.4)	1.20 (1.0–1.4)	1.13 (1.0–1.3)	1.18 (1.0–1.4)	1.23 (1.1–1.4)	1.22 (1.0–1.4)	1.19 (1.0–1.4)	1.17 (1.0–1.4)
ICCAD	3347	7.72 (7.2–8.3)	7.82 (7.2–8.4)	7.55 (7.1–8.0)	7.73 (7.2–8.3)	7.82 (7.2–8.4)	7.85 (7.3–8.4)	7.71 (7.2–8.3)	7.64 (7.2–8.2)

Abbreviations: *n*: number of subjects; BMI: body mass index; ALT: alanine aminotransferase; TC: total cholesterol; TG: triglycerides; HDL-C: high-density lipoprotein cholesterol; LDL-C: low-density lipoprotein cholesterol; C-IMT: carotid intima-media thickness; ICCAD: inter-adventitia common carotid artery diameter. Unit of measure: ALT: U/L; BMI: kg/m^2^; TC, LDL-C, TG, and glucose: mmol/L.

**Table 2:** Association of rs738409 (C/G), rs10401969 (T/C), and rs1260326 (C/T) with c-IMT measures and ICCAD.

Ultrasonographic Measures	*n*	SNP	EA	Model 1			Model 2		
				β	SE	*p*	β	SE	*p*
c-IMT_mean_	3346	rs738409	G	−0.002	0.0029	0.443	−0.001	0.003	0.818
		rs10401969	C	0.012	0.005	0.025	0.001	0.004	0.768
		rs1260326	T	−0.004	0.002	0.060	0.003	0.002	0.179
c-IMT_max_	3346	rs738409	G	0	0.006	0.963	0.002	0.005	0.636
		rs10401969	C	0.014	0.009	0.108	0	0.008	0.970
		rs1260326	T	−0.01	0.004	0.013	0.001	0.004	0.810
c-IMT_mean-max_	3347	rs738409	G	−0.003	0.003	0.353	−0.001	0.003	0.663
		rs10401969	C	0.014	0.005	0.006	0.003	0.005	0.476
		rs1260326	T	−0.005	0.002	0.02	0.003	0.002	0.176
ICCAD	3347	rs738409	G	0	0.001	0.499	0.002	0.001	0.174
		rs10401969	C	0.006	0.002	0.015	0.001	0.002	0.732
		rs1260326	T	−0.005	0.001	<0.001	−0.001	−0.001	0.271

Abbreviations: *n*: number of subjects; EA: effect allele; SNP: single nucleotide polymorphism. Model 1 univariate analysis. Model 2 adjusted for age, sex, and multidimensional scaling dimensions (MSD) 1–3. All c-IMT variables and ICCAD were logarithmically transformed before statistical analysis.

**Table 3:** Association of the effect allele at rs738409 (C/G), rs10401969 (T/C), and rs1260326 (C/T) with metabolic traits.

Metabolic Traits	*n*	SNP	EA	Model 1			Model 2		
				β	SE	*p*	β	SE	*p*
ALT	3347	rs738409	G	0.031	0.006	<0.001	0.029	0.006	<0.001
		rs10401969	C	0.042	0.01	<0.001	0.040	0.009	<0.001
		rs1260326	T	−0.012	0.004	0.009	−0.008	0.004	0.062
BMI	3346	rs738409	G	0.296	0.136	0.029	0.265	0.132	0.045
		rs10401969	C	0.181	0.225	0.421	−0.038	0.219	0.816
		rs1260326	T	−0.569	0.101	<0.001	−0.224	0.102	0.028
TC	3341	rs738409	G	−0.025	0.036	0.489	−0.026	0.034	0.441
		rs10401969	C	−0.13	0.059	0.028	−0.042	0.057	0.465
		rs1260326	T	0.122	0.027	<0.001	0.027	0.027	0.312
LDL-C	3347	rs738409	G	−0.008	0.032	0.803	−0.007	0.031	0.831
		rs10401969	C	−0.096	0.053	0.069	−0.022	0.051	0.659
		rs1260326	T	0.064	0.024	0.008	−0.027	0.024	0.255
TG	3341	rs738409	G	−0.018	0.026	0.474	−0.022	0.025	0.388
		rs10401969	C	−0.044	0.042	0.300	−0.030	0.042	0.482
		rs1260326	T	0.114	0.019	<0.001	0.105	0.020	<0.001
Glucose	2426	rs738409	G	0.024	0.025	0.344	0.027	0.023	0.233
		rs10401969	C	0.087	0.041	0.035	0.033	0.038	0.376
		rs1260326	T	−0.087	0.018	<0.001	−0.031	0.017	0.076

Abbreviations: *n*: number of subjects; EA: effect allele; ALT: alanine aminotransferase; BMI: body mass index; TC: total cholesterol; LDL-C: low-density lipoprotein cholesterol; TG: triglycerides. Unit of measure: ALT: U/L; BMI: kg/m^2^; TC, LDL-C, TG, and glucose: mmol/L. Model 1: univariate analysis; Model 2: adjusted by age, sex, and MDS 1–3. ALT and TG were logarithmically transformed before statistical analysis because of skewed distributions.

## Supplementary Materials

The Supplementary Materials are available online https://www.mdpi.com/2073-4425/11/11/1243/s1, Table S1: Distribution of c-IMT and ICCAD measures across rs738409 (C/G), rs10401969 (T/C), and rs1260326 (C/T) genotype groups in the different ALT quartiles, Table S2: Association of rs10401969 (T/C) with measures of c-IMT and ICCAD in the different ALT quartiles.
