# Supplementary material for: Genetic Variants Associated with Non-Alcoholic Fatty Liver Disease Do Not Associate with Measures of Sub-Clinical Atherosclerosis: Results from the IMPROVE Study
Source: Genes (Basel). 2020 Oct 22;11(11):1243. doi: 10.3390/genes11111243 (PMC7690395; doi:10.3390/genes11111243)
Supplement: Supplementary file 1 [file genes-11-01243-s001.pdf]

Supplementary Material

Genetic variants associated with NAFLD do not associate with measures of sub-clinical atherosclerosis. Results from the IMPROVE study

Luigi Castaldo[1,2,*], Federica Laguzzi[3], Rona J. Strawbridge[4,5,6], Damiano Baldassarre[7,8], Fabrizio Veglia[7], Lorenzo Vigo[7], Elena Tremoli[7], Ulf de Faire[3], Per Eriksson[6], Andries Smit[9], Jiri Aubrecht[10], Karin Leander[3], Matteo Pirro[11], Philip Giral[12], Alberto Ritieni[2], Giovanni Di Minno[1], Anders Mälarstig[6] & Bruna Gigante[6], on behalf of IMPROVE study group.

[1]Department of Clinical Medicine and Surgery, University of Naples "Federico II", Naples, Italy,
[2]Department of Pharmacy, University of Naples "Federico II", Naples, Italy,
[3]Unit of Cardiovascular and Nutritional Epidemiology, Institute of Environmental Medicine, Karolinska Institutet
[4]Mental Health and Wellbeing, Institute of Health and Wellbeing, University of Glasgow Glasgow, UK
[5]Health Data Research UK
[6]Cardiovascular Medicine, Dept of Medicine, Karolinska Institutet, Stockholm, Sweden.
[7]Centro Cardiologico Monzino, IRCCS, Milan, Italy
[8]Department of Medical Biotechnology and Translational Medicine, University of Milan, Milan, Italy
[9]Department of Medicine, Division of vascular medicine University Medical Center Groningen, Groningen, The Netherlands
[10]Takeda Pharmaceuticals International Co., Cambridge, Massachusetts, USA.
[11]Unit of Internal Medicine, Department of Medicine, University of Perugia, Perugia, Italy
[12]Unités de Prévention Cardiovasculaire, Assistance Publique-Hôpitaux de Paris, Service Endocrinologie-Metabolisme, Groupe Hôpitalier Pitie-Salpetriere, Paris, France
[*]luigi.castaldo2@unina.it

1. Complete list of IMPROVE study group

- *Dipartimento di Scienze Farmacologiche e Biomolecolari, Università di Milano, Milan, Italy:* C.R. Sirtori, S. Castelnuovo, L. Calabresi.

- *Centro Cardiologico Monzino, IRCCS, Milan Italy:* M. Amato, B. Frigerio, A. Ravani, D. Sansaro, D. Coggi, CC. Tedesco, A. Bonomi,

- *Cardiovascular Medicine Unit. Dept of Medicine Karolinska Institutet and Karolinska University Hospital Solna, Sweden:* A. Silveira, L. Nilsson, P. Fahlstadius.

- *University College of London, Department of Medicine, Rayne Institute, London, United Kingdom:* J. Cooper, J. Acharya, S.E. Humphries.

- *Foundation for Research in Health Exercise and Nutrition, Kuopio Research Institute of Exercise Medicine, Kuopio, Finland:* K. Huttunen, E. Rauramaa, H Pekkarinen, I.M. Penttila, J. Törrönen, R. Rauramaa.

- *Department of Medicine, University Medical Center Groningen, Groningen & Isala Clinics Zwolle, Department of Medicine; the Netherlands:* A.I. van Gessel, A.M van Roon, G.C. Teune, W.D. Kuipers, M. Bruin, A. Nicolai, P. Haarsma-Jorritsma, D.J. Mulder, H.J.G. Bilo, G.H. Smeets, A.J. Smit.

- *Assistance Publique - Hôpitaux de Paris; Service Endocrinologie-Metabolisme, Groupe Hôpitalier Pitié-Salpetriere, Unités de Prévention Cardiovasculaire, Paris, France:* J.L. Beaudeux, J.F. Kahn, V. Carreau, A. Kontush, P. Giral.

- *Institute of Public Health and Clinical Nutrition, University of Eastern Finland, Kuopio Campus:* J. Karppi, T. Nurmi, K. Nyyssönen, R. Salonen, T.P. Tuomainen, , J.Tuomainen, J. Kauhanen, S. Kurl.

- *Internal Medicine, Angiology and Arteriosclerosis Diseases, Department of Clinical and Experimental Medicine, University of Perugia, Perugia, Italy*: G. Vaudo, A. Alaeddin, D. Siepi, G. Lupattelli, E. Mannarino.

2. Ethics

The study was designed in accordance with the rules of Good Clinical Practice, and with the ethical principles established in the Declaration of Helsinki. Each participant provided two different informed consents; one for general participation in the study and one for genotyping. The study was approved by the Institutional Review Board (IRB) at each one of the seven recruiting centers: (1) the Regional Ethics Review Board at Karolinska Institutet, Stockholm Sweden, (2) IRB at the Groupe Hôpitalier Pitie-Salpetriere, Paris, France, (3) the IRB Comitato Etico delle Aziende Sanitarie della regione Umbria, Perugia and (4) the IRB at the Ospedale Niguarda Ca´Granda, Milano, both in Italy, (5) the IRB at the University Hospital Groningen, Groningen, the Netherlands, (6) the IRB Hospital District of Northern Savo and (7) and the IRB at University of Eastern Finland, both in Kuopio, Finland. Each participant provided two different written consents one for general participation in the study and one for genotyping.

Table list

Supplementary Table S1. Distribution of c-IMT and ICCAD measures across rs738409 (C/G), rs10401969 (T/C), and rs1260326 (C/T) genotype groups in the different ALT quartiles.

Supplementary Table S2. Association of rs10401969 (T/C) with measures of c-IMT and ICCAD in the different ALT quartiles.

Supplementary Table S1. Distribution of c-IMT and ICCAD measures across rs738409 (C/G), rs10401969 (T/C), and rs1260326 (C/T) genotype groups in the different ALT quartiles.

**Quartile**	***n***		**rs738409**			**rs10401969**		**rs1260326**		
			**CC**	**CG**	**GG**	**TT**	**CT+CC**	**CC**	**CT**	**TT**
			**(*n*=623)**	**(*n*=227)**	**(*n*=26)**	**(*n*=786)**	**(*n*=90)**	**(*n*=265)**	**(*n*=433)**	**(*n*=178)**
I	876	c-IMT$_{mean}$	0.85	0.87	0.78	0.85	0.85	0.86	0.85	0.83
			(0.74-0.99)	(0.73-1.01)	(0.73-0.95)	(0.74-1.00)	(0.70-0.97)	(0.75-0.99)	(0.75-1.00)	(0.72-0.98)
		c-IMT$_{max}$	1.85	1.94	1.8	1.88	1.84	1.93	1.85	1.85
			(1.45-2.55)	(1.45-2.61)	(1.39-2.31)	(1.45-2.59)	(1.39-2.42)	(1.45-2.55)	(1.45-2.59)	(1.39-2.41)
		c-IMT$_{mean\text{-}max}$	1.21	1.22	1.13	1.21	1.22	1.23	1.22	1.17
			(1.04-1.41)	(1.04-1.48)	(1.00-1.36)	(1.04-1.42)	(1.04-1.41)	(1.05-1.42)	(1.05-1.42)	(1.02-1.42)
		ICCAD	7.68	7.8	7.27	7.68	7.81	7.79	7.64	7.64
			(7.17-8.27)	(7.20-8.30)	(6.92-7.89)	(7.15-8.24)	(7.22-8.34)	(7.20-8.25)	(7.15-8.30)	(7.10-8.15)
			**CC**	**CG**	**GG**	**TT**	**CT+CC**	**CC**	**CT**	**TT**
			**(*n*=632)**	**(*n*=274)**	**(*n*=23)**	**(*n*=827)**	**(*n*=102)**	**(*n*=279)**	**(*n*=413)**	**(*n*=237)**
II	929	c-IMT$_{mean}$	0.85	0.85	0.88	0.85	0.84	0.86	0.85	0.84
			(0.74-0.98)	(0.76-0.99)	(0.74-1.04)	(0.75-0.98)	(0.74-1.03)	(0.74-1.02)	(0.75-0.97)	(0.74-0.98)
		c-IMT$_{max}$	1.85	1.88	1.93	1.85	1.84	1.85	1.85	1.85
			(1.45-2.50)	(1.39-2.42)	(1.48-2.42)	(1.45-2.42)	(1.39-2.61)	(1.45-2.61)	(1.48-2.42)	(1.39-2.41)
		c-IMT$_{mean\text{-}max}$	1.18	1.16	1.22	1.18	1.2	1.2	1.18	1.16
			(1.05-1.39)	(1.05-1.41)	(1.00-1.54)	(1.05-1.39)	(1.07-1.45)	(1.05-1.45)	(1.05-1.37)	(1.03-1.38)
		ICCAD	7.67	7.79	7.89	7.69	7.8	7.81	7.69	7.6
			(7.18-8.24)	(7.28-8.34)	(7.11-8.30)	(7.21-8.27)	(7.22-8.38)	(7.27-8.35)	(7.21-8.27)	(7.17-8.24)
			**CC**	**CG**	**GG**	**TT**	**CT+CC**	**CC**	**CT**	**TT**
			**(*n*=546)**	**(*n*=235)**	**(*n*=20)**	**(*n*=691)**	**(*n*=110)**	**(*n*=253)**	**(*n*=373)**	**(*n*=175)**
III	801	c-IMT$_{mean}$	0.85	0.86	0.87	0.85	0.87	0.86	0.85	0.85
			(0.74-0.98)	(0.73-1.01)	(0.75-0.94)	(0.73-1.00)	(0.76-1.03)	(0.74-1.00)	(0.73-0.99)	(0.74-1.02)

		c-IMT$_{max}$	1.84	1.85	1.8	1.84	1.85	1.85	1.76	1.85
			(1.39-2.41)	(1.39-2.51)	(1.42-2.59)	(1.39-2.41)	(1.48-2.51)	(1.45-2.5)	(1.39-2.32)	(1.35-2.5)
		c-IMT$_{mean-max}$	1.2	1.18	1.2	1.19	1.23	1.22	1.18	1.2
			(1.02-1.42)	(1.03-1.41)	(1.07-1.33)	(1.02-1.40)	(1.06-1.49)	(1.03-1.42)	(1.02-1.38)	(1.03-1.45)
		ICCAD	7.68	7.78	7.8	7.73	7.78	7.84	7.64	7.64
			(7.19-8.33)	(7.22-8.29)	(7.33-8.12)	(7.20-8.27)	(7.20-8.46)	(7.31-8.48)	(7.14-8.16)	(7.21-8.29)
			**CC**	**CG**	**GG**	**TT**	**CT+CC**	**CC**	**CT**	**TT**
			**(*n*=453)**	**(*n*=249)**	**(*n*=39)**	**(*n*=631)**	**(*n*=110)**	**(*n*=273)**	**(*n*=330)**	**(*n*=178)**
IV	741	c-IMT$_{mean}$	0.84	0.85	0.78	0.83	0.88	0.86	0.85	0.82
			(0.73-0.99)	(0.76-1.00)	(0.71-0.88)	(0.73-1.00)	(0.78-1.01)	(0.76-1.00)	(0.73-1.02)	(0.74-0.95)
		c-IMT$_{max}$	1.84	1.94	1.67	1.78	2.13	1.93	1.85	1.74
			(1.36-2.50)	(1.45-2.59)	(1.20-2.22)	(1.35-2.50)	(1.67-2.61)	(1.45-2.51)	(1.35-2.59)	(1.39-2.31)
		c-IMT$_{mean-max}$	1.19	1.23	1.1	1.18	1.26	1.23	1.19	1.16
			(1.02-1.40)	(1.04-1.44)	(0.98-1.28)	(1.01-1.39)	(1.11-1.48)	(1.05-1.43)	(1.01-1.44)	(1.02-1.36)
		ICCAD	7.87	7.92	7.56	7.87	7.84	7.97	7.8	7.72
			(7.34-8.44)	(7.33-8.46)	(7.11-8.09)	(7.32-8.42)	(7.35-8.51)	(7.35-8.56)	(7.34-8.41)	(7.21-8.31)

Data are reported as median and IQR. Minor allele frequency (%) for each quartile (Q): rs738409-G Q1:16, Q2:17, Q3:17, Q4:22; rs10401969-C Q1:5, Q2:5, Q3:7, Q4:8; rs1260326-T Q1:45, Q2:47, Q3:45, Q4:44. Abbreviations: *n* number of subjects

Supplementary Table S2. Association of rs10401969 (T/C) with measures of c-IMT and ICCAD in the different ALT quartiles.

**ù**	***n***	**Ultrasonographic measures**	**Model 1**			**Model 2**		
			**β**	**SE**	***p***	**β**	**SE**	***p***
I	876	c-IMT $_{mean}$	0.003	0.013	0.765	-0.004	0.009	0.667
		c-IMT $_{max}$	-0.01	0.019	0.574	-0.021	0.011	0.224
		c-IMT $_{mean-max}$	0.002	0.011	0.847	-0.006	0.01	0.559
		ICCAD	0.005	0.005	0.309	0.002	0.004	0.697
II	929	c-IMT $_{mean}$	0.003	0.009	0.704	-0.008	0.008	0.317
		c-IMT $_{max}$	0	0.168	1	-0.015	0.016	0.33
		c-IMT $_{mean-max}$	0.006	0.01	0.558	-0.007	0.009	0.432
		ICCAD	0.005	0.005	0.275	-0.001	0.004	0.866
III	801	c-IMT $_{mean}$	0.012	0.009	0.177	0.003	0.008	0.756
		c-IMT $_{max}$	0.015	0.017	0.378	-0.002	0.016	0.894
		c-IMT $_{mean-max}$	0.016	0.01	0.092	0.005	0.009	0.575
		ICCAD	0.005	0.005	0.254	0	0.004	0.958
IV	741	c-IMT $_{mean}$	0.024	0.01	0.015	0.016	0.009	0.077
		c-IMT $_{max}$	0.051	0.018	0.004	0.039	0.016	0.018
		c-IMT $_{mean-max}$	0.031	0.01	0.003	0.022	0.009	0.021
		ICCAD	0.006	0.005	0.227	0.001	0.004	0.769

Abbreviations: *n* number of subjects. Model 1 univariate analysis
Model 2 adjusted for age, sex and population structure All c-IMT variables and ALT were logarithmically transformed before statistical analysis
